# Wide-Temperature-Range Large-Deformation Tensile Behavior and Constitutive Modeling of Polytetrafluoroethylene

**DOI:** 10.3390/ma19183827

**Published:** 2026-09-08

**Authors:** Lushan Li, Le Chang, Jianping Zhao, Xiaowei Wang, Jizhong Yan, Zechen Yang, Shengping Wu

**Affiliations:** 1 School of Mechanical and Power Engineering, Nanjing Tech University, Nanjing 211816, China; 2 Jiangsu Special Equipment Safety Supervision and Inspection Institute, Nanjing 210036, China; 3 Jiangsu Province Market Supervision Technology Innovation Center (Safe Operation and Maintenance of Oil and Gas Storage and Transportation Facilities), Nanjing 211162, China

**Keywords:** polytetrafluoroethylene, mechanical properties, constitutive model, molecular dynamics simulation

## Abstract

This study investigates the tensile behavior of polytetrafluoroethylene (PTFE) tube material across a wide range of temperatures, displacement rates, and sampling directions. Temperature has a dominant effect: flow stress decreases and ductility increase markedly as temperature rises, while displacement rate and sampling direction show only minor effects. A temperature-coupled three-part superposition quasi-static stress (TPS) model is developed, using Arrhenius-type relationships to describe how the model parameters evolve with temperature. This model outperforms the Johnson–Cook, Ogden, and Zhu–Wang–Tang (ZWT) models across the full strain range and the entire tested temperature range. Molecular dynamics simulations further show that rising temperature increases free volume and chain-segment mobility while reducing chain orientation, providing a molecular-scale explanation for the observed thermal softening.

## 1. Introduction

Polytetrafluoroethylene, PTFE, is widely used in petrochemical, semiconductor manufacturing, steel, and power industries because of its excellent chemical inertness, outstanding temperature resistance, and extremely low friction coefficient [1,2,3]. PTFE expansion joints serve as key components in piping systems. Their primary function is to absorb pipeline displacement and additional stress caused by temperature change and mechanical vibration, thereby ensuring the pressure-bearing safety and stable operation of the entire piping system. As industrial operating conditions become increasingly demanding, PTFE expansion joints now face service environments characterized by wide temperature ranges and large deformation. These extreme conditions place higher requirements on the mechanical performance of the material.

Prior studies have extensively explored PTFE-modified composite systems and their microstructures [4,5,6]. Filler modification and drawing processes have been shown to significantly affect the mechanical strength and dimensional stability of the material [7]. The macroscopic stress response of PTFE and its modified variants typically shows three distinct stages, and this macroscopic behavior is closely tied to the evolution of the underlying crystalline structure.

As a typical nonlinear polymer, PTFE exhibits pronounced temperature sensitivity and nonlinearity in its mechanical behavior. Under different temperatures and loading conditions, the stress–strain relationship changes dramatically, and this complexity resists accurate description by simple classical theories. Establishing a constitutive model with clear physical meaning, complete mathematical form, and a wide temperature range of applicability is therefore essential. Such a model would guide the engineering design of PTFE expansion joints and support the accurate prediction of their service life. It bears directly on structural safety and reliability, and it also carries theoretical significance for polymer materials science. In engineering practice, the ZWT model [8] incorporates a Maxwell element and has performed well in capturing rate dependence for a range of polymers. Zhang et al. [9] introduced a higher-order equilibrium stress correction and proposed the three-part superposition quasi-static stress model, known as TPS. This model, however, was developed mainly for low-temperature applications. Its applicability across the range from room temperature to high temperature, particularly near the softening temperature of PTFE, has not been thoroughly validated. Because PTFE must maintain stable and reliable mechanical performance over a wide temperature range in actual service, a constitutive model with broader temperature applicability and higher predictive accuracy is urgently needed.

This study addresses these needs through four connected efforts. First, systematic uniaxial tensile tests were performed on PTFE under different sampling directions, displacement rates, and temperatures, yielding true stress–strain curves and mechanical property parameters across a wide temperature range and large deformation. These results provide the experimental basis for constitutive model development and parameter calibration. Second, the ability of the classical Johnson–Cook, Ogden, and ZWT models to describe the tensile response of PTFE was evaluated, and their applicability and limitations in capturing the yield plateau, large-deformation strain hardening, and temperature-dependent response were analyzed. Third, building on Arrhenius thermal activation theory and the original TPS framework, temperature-dependent evolution relationships were introduced for the phenomenological parameters, establishing a temperature-coupled TPS constitutive model applicable from room temperature to high temperature. Fourth, molecular dynamics simulations were conducted to examine how the free volume and segmental mobility of PTFE evolve with temperature, providing a molecular-scale basis for interpreting the macroscopic mechanical response and the temperature effects embedded in the constitutive model.

## 2. Materials and Methods

### 2.1. Uniaxial Tensile Testing

**Figure 1:**
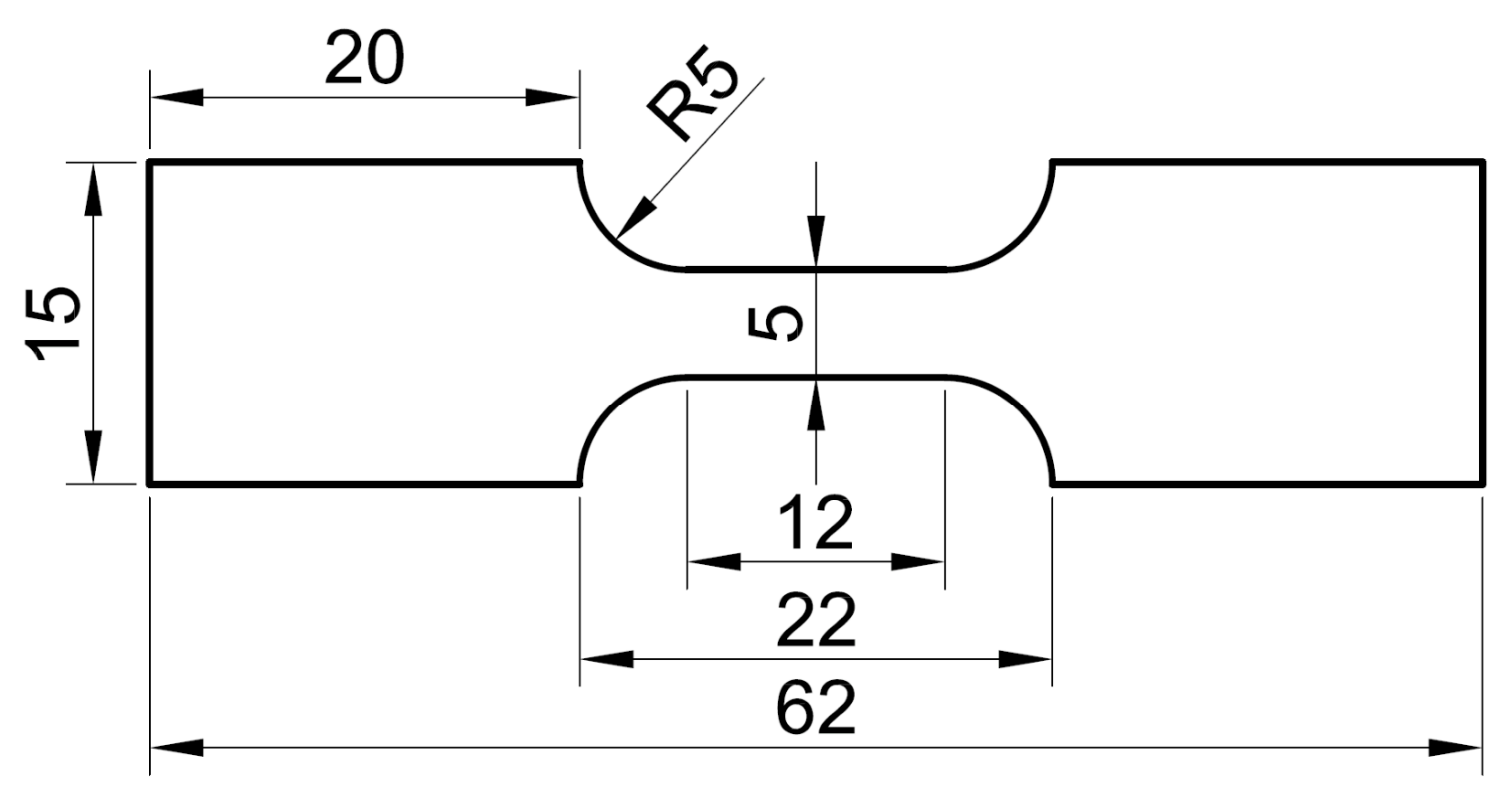
Uniaxial tensile specimen.

The PTFE tubing used for the test samples was provided by Jiangyin Jiangnan Fluoroplastics Co., Ltd., Wuxi, China, and was manufactured using sintering molding. The original tubing had an outer diameter of 275 mm and a wall thickness of 13 mm. Tensile specimens were machined according to the standard QB/T 4877-2015 [10], Polytetrafluoroethylene Tube, with the specimen dimensions shown in Figure 1. The dumbbell-shaped specimens were machined from the PTFE pipe wall at room temperature using precision machining, without any thermal cutting process, thus eliminating the heat-affected zone. The specimen thickness and gauge length are 3 mm and 12 mm, respectively. Tensile tests were performed using the IPBF-5000 planar biaxial mechanical testing system developed by CARE Corporation, Tianjin, China. The maximum loading range of this testing machine is 5 KN, the load accuracy can be controlled within ±0.4% of the full scale, the displacement measurement accuracy is ±0.01 mm, and the data acquisition frequency is 10 Hz. By transferring the instrumental accuracy to the stress and strain calculations, the relative uncertainties of engineering stress and strain at the strain level reported in this study were estimated to be less than 2%. Therefore, the differences between samples, rather than the instrumental accuracy, are the main source of uncertainty in the reported mechanical parameters. The high-temperature test was conducted in a resistance heating environmental chamber integrated with the IPBF-5000 test system, equipped with a closed-loop PID temperature controller, and thermocouples were installed near the gauge length of the specimen. The temperature control accuracy at the set temperature is ±1 °C. Before loading, the specimen is held at the target temperature for 10 min. Preliminary tests confirmed that, considering the small cross-section of the specimen, this holding time is sufficient to allow the temperature of the specimen core to reach equilibrium with the temperature inside the chamber.

To obtain both engineering and real stress–strain responses without relying on the constant volume assumption, this study employed a dual-camera stereo digital image correlation (DIC) system during testing on a testing machine. Random speckle patterns were prepared on the surface of the specimen along its gauge length, and two simultaneous cameras recorded the full-field displacement of the specimen surface throughout the tensile process. Engineering strain was obtained using a virtual extensometer, measured between two tracking points with an initial gauge length of $L_{0}$. The DIC displacement field provides the instantaneous distance L between these two points at each time step, and the engineering strain is calculated as follows:

(1) $$\varepsilon_{eng}\;=\frac{\left(L-L_{0}\;\right)}{L_{0}}$$

The engineering stress is calculated by dividing the load F synchronously recorded by the force sensor by the initial cross-sectional area $A_{0}$:

(2) $$\sigma_{eng\;}=\frac{F}{A_{0}}$$

The DIC displacement field simultaneously provides the true strain $\varepsilon_{22}$ in the width direction and the true strain $\varepsilon_{33}$ in the thickness direction. Based on this, the true stress can be directly calculated from the instantaneous cross-sectional area without assuming that the volume remains constant:

(3) $$A=A_{0}\;exp(\varepsilon_{22}\;+\varepsilon_{33}\;)$$

(4) $$\sigma_{true}\;=\frac{F}{A}$$

The axial true strain is calculated as follows:

(5) $$\varepsilon_{true}\;=ln(1+\varepsilon_{eng}\;)$$

To investigate the effects of sampling direction and displacement rate on the tensile properties of PTFE, specimens were taken along the circumferential and longitudinal directions of the tube. Tests were conducted at 300.15 K with displacement rates of 20, 50, and 100 mm/min. Six groups of tests were performed in total, with the conditions listed in Table 1. The elastic modulus is taken as the slope of the initial small strain segment of the engineering stress–strain curve. The yield strength is taken as the engineering stress corresponding to the inflection point where the curve deviates from the initial linear segment and enters the yield plateau. The tensile strength is defined as the maximum engineering stress recorded throughout the entire measured strain range. The fracture elongation at break is taken as the engineering strain corresponding to the moment the specimen fractures.

### 2.2. Molecular Dynamics Simulation Methodology

A semicrystalline PTFE initial model was constructed using a custom Python (Version: 3.9) script. The repeat unit of the PTFE chain is CF_2_CF_2_. The system consists of 80 molecular chains, each containing 50 repeat units, for a total of 24,000 atoms. Seventy percent of the chains are arranged in the pseudo-hexagonal Phase I configuration to form the crystalline region, and the remaining thirty percent are generated as amorphous conformations through a random walk algorithm and packed into the interstitial spaces of the crystalline region [11]. The crystalline and amorphous regions alternate under periodic boundary conditions to form a semicrystalline structure, with an initial model density of about 2.02 g/cm^3^ [12]. Intramolecular and intermolecular interactions are described using the OPLS-AA perfluoroalkane force field [13,14], with non-bonded interactions represented by a Lennard–Jones 12-6 potential and a cutoff radius of 10 Å. Bond length, bond angle, and dihedral angle parameters were validated through prior energy minimization and density correction, and they stably maintain the helical conformation of the PTFE chain.

All simulations were performed using LAMMPS (Version: 22 July 2025) [15], with real units and three-dimensional periodic boundary conditions. The initial model underwent two stages of energy minimization, followed by NVT annealing to remove internal stress introduced during construction, and multi-stage NPT compaction at 600 K to correct the system density. NPT equilibration and NVT production runs were then performed separately at 300.15, 323.15, 373.15, 423.15, and 473.15 K for analysis of free volume and segmental mobility. Based on the equilibrated configurations at two representative temperatures, 300.15 and 473.15 K, uniaxial tensile simulations were performed under the NVT ensemble at an engineering strain rate of 1.5 × 10^9^ s^−1^ up to a maximum engineering strain of 150 percent. This strain rate is considerably higher than the rates corresponding to the 20 to 100 mm/min used in the experiments, a common practice given the timescale limitations of molecular dynamics [16,17].

The free volume was calculated using the radical Voronoi tessellation [18], a well-established approach for handling polydisperse atomic systems. Carbon and fluorine atoms were assigned van der Waals radii consistent with the OPLS-AA force field parameterization used for non-bonded interactions in this study. The fluorine atom radius was set smaller than that of the carbon atom, in agreement with standard van der Waals radius values. Atoms in the crystalline and amorphous regions distinguished by the Q6 order parameter and analyzed separately. Segmental mobility was characterized through the mean-square displacement of the chain centroid, MSD(t), and the diffusion coefficient was extracted from the long-time linear region using the Einstein relation *D* = MSD(t)/(6t). The apparent activation energy was obtained by fitting the Arrhenius equation *D* = *D*_0_exp(−*Q/RT*). The degree of chain orientation was described using the Herman orientation factor *f* = (3⟨cos^2^θ⟩ − 1)/2 [19], where θ is the angle between the principal axis of the molecular gyration tensor and the tensile direction. A value of *f* = 1 corresponds to chains fully aligned with the tensile direction, and *f* = 0.5 corresponds to chains fully perpendicular to it. Crystallinity was determined atom by atom based on the Steinhardt Q6 bond orientational order parameter [20], with atoms with Q6 greater than 0.283 classified as crystalline, and its evolution was tracked across seven strain points spanning 0 to 150 percent strain.

## 3. Results and Discussion

### 3.1. Tensile Mechanical Properties

**Figure 2:**
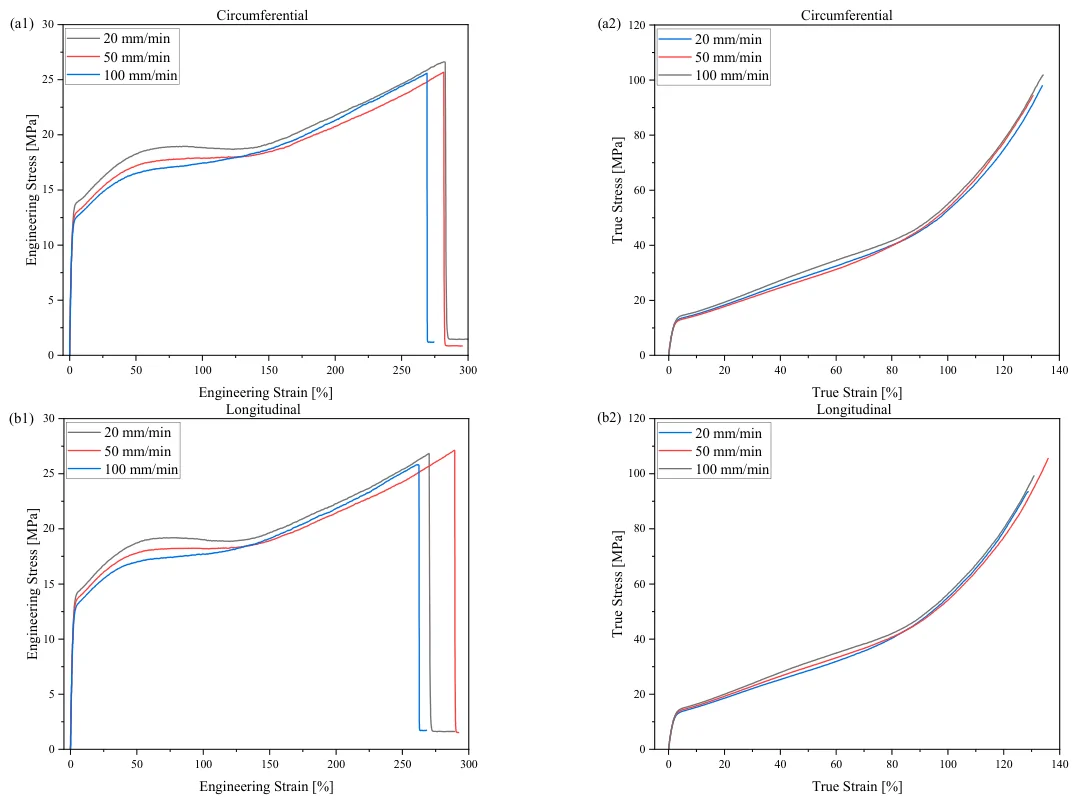
Engineering and true stress–strain curves of PTFE under different displacement rates: (**a1**,**a2**) are circumferential specimens; (**b1**,**b2**) are longitudinal specimens.

Figure 2 and Table 2 present the stress–strain curves and mechanical property parameters of PTFE under different displacement rates and sampling directions. Due to the limited quantity of PTFE pipes available for this study, three samples were tested for each operating condition at 300.15 K, while only one sample was tested for each operating condition at high temperature. The values listed in Table 2 are the average values and their upper and lower error ranges for the corresponding operating conditions. Together, these results show that the material exhibits low strain-rate sensitivity and macroscopic isotropy over the tested range. At a given displacement rate, the elastic modulus, yield strength, tensile strength, and fracture elongation of the circumferential and longitudinal specimens are nearly identical. It was demonstrated that no significant macroscopic anisotropy of tensile properties was observed under the studied conditions, indicating that the crystalline grains and amorphous polymer chains are randomly distributed in three dimensions without significant processing-induced orientation [21]. As the displacement rate increases from 20 to 100 mm/min, the yield strength in both directions rises slightly. The circumferential yield strength increases from $12.73_{-0.23}^{+0.18}$ MPa to $13.75_{-0.40}^{+0.30}$ MPa, and the longitudinal yield strength increases from $13.36_{-0.30}^{+0.22}$ MPa to $13.79_{-0.13}^{+0.19}$ MPa. Tensile strength shows a similar positive rate dependence. This behavior arises because a higher loading rate shortens the relaxation time available for polymer chain segments to respond to the applied force. The chains cannot fully release internal stress through slippage and disentanglement, so the macroscopic resistance to deformation increases [22].

Across all test conditions, the stress–strain curves follow the three-stage deformation pattern typical of polymers. After the initial elastic stage and yield plateau, the material enters a large-deformation uniform extension stage. Once the true strain exceeds about 80%, the polymer chains progressively align along the tensile direction and the crystalline regions rearrange [23]. The curve slope then increases and the material shows clear strain hardening. The engineering fracture elongations recorded in Table 2 range from $263_{-2}^{+3}$% to $281_{-4}^{+4}$%, at which point the macromolecular chains reach their limiting orientation. This agreement shows that varying the displacement rate does not significantly affect the extensibility of the material or its room-temperature fracture mechanism.

To further examine the effects of sampling direction and temperature on the tensile properties of PTFE, specimens taken along the circumferential and longitudinal directions were tested at 323.15, 373.15, 423.15, and 473.15 K, with a displacement rate of 50 mm/min. Eight groups of tests were performed in total, with the conditions listed in Table 3.

**Figure 3:**
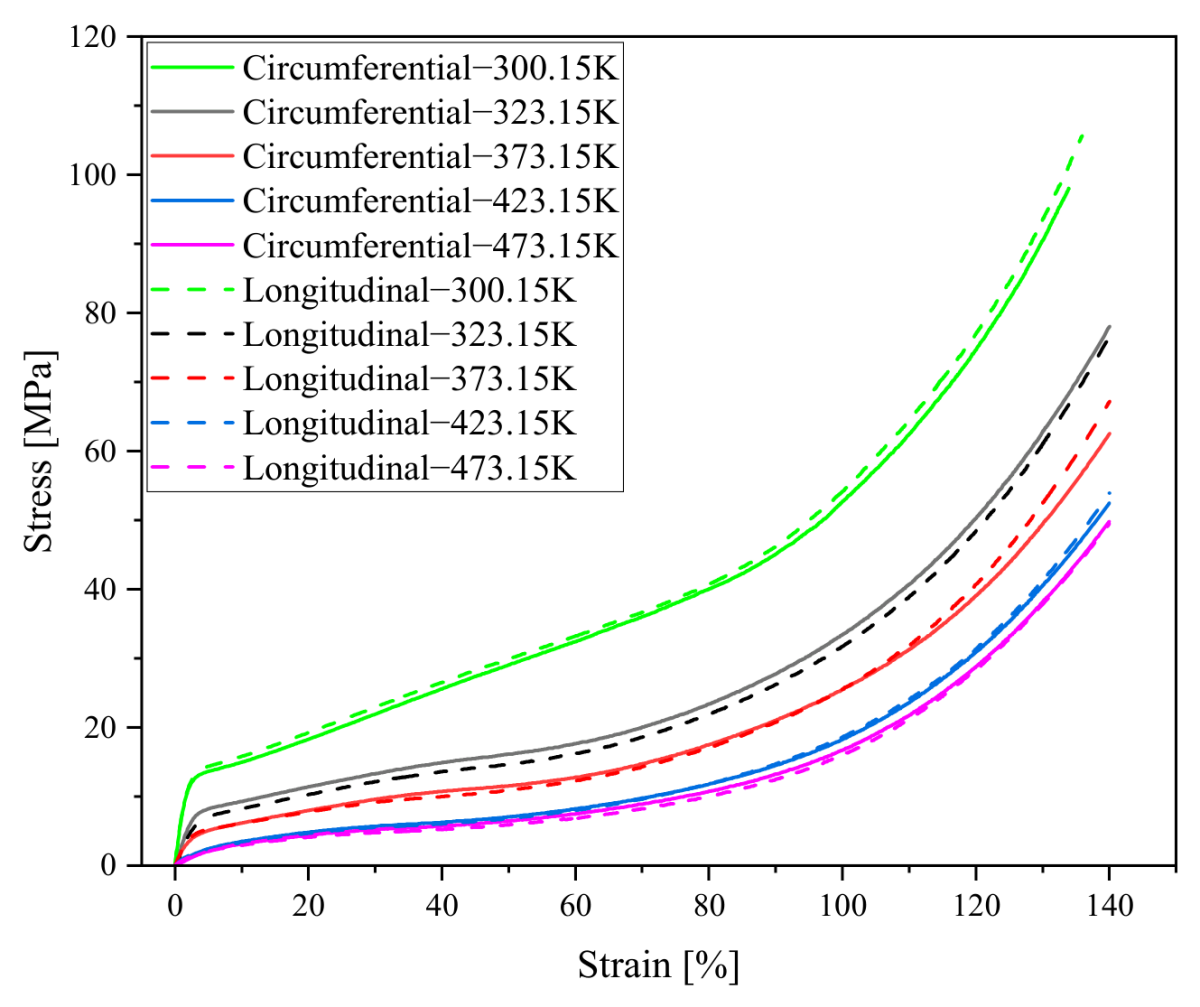
True stress–strain curves of PTFE at different test temperatures and sampling directions.

The true stress–strain curves of PTFE at different test temperatures and sampling directions are shown in Figure 3. The material displays strong temperature sensitivity and retains clear macroscopic isotropy across all temperatures. As the test temperature rises from 300.15 to 473.15 K, the free volume within the polymer matrix increases and the thermal motion of the chain segments intensifies, weakening the interchain interaction forces [24]. The curves shift markedly downward, and the flow stress at a given strain decreases substantially with increasing temperature. When the temperature exceeds 423.15 K, the elastic segment of the true stress–strain curve nearly disappears. The flow stress decreases only slightly further at 473.15 K, indicating that the material undergoes pronounced softening above 423.15 K. In terms of fracture behavior, specimens tested at temperatures above 300.15 K remained intact even after deformation exceeded 300%, which reached the maximum stroke of the testing machine; consequently, the fracture strain could not be measured. This shows that increasing temperature markedly enhances the ductility of PTFE.

### 3.2. Evaluation of Existing Constitutive Models

Having established the experimental tensile behavior of PTFE across the tested temperature and strain-rate ranges in Section 3.1, this section evaluates whether existing constitutive models can adequately describe this behavior, thereby identifying the specific requirements that a new model must satisfy. An ideal constitutive model should capture the full stress–strain response, from small-strain elastic deformation to large-deformation strain hardening, and should also represent temperature effects reliably. This study therefore follows a systematic approach of evaluating existing models broadly, identifying their limitations, and then developing a targeted improvement. The Johnson–Cook and Ogden models are assessed first, the applicability boundaries of the classical ZWT model are then examined, and, finally, a temperature-coupled TPS model incorporating thermomechanical coupling is constructed.

The Johnson–Cook model is a phenomenological constitutive model widely applied in the dynamic mechanical analysis of metals and reactive composites. Its mathematical form is simple, its parameters carry clear physical meaning, and it can describe strain hardening, strain-rate strengthening, and thermal softening within a unified framework [25]. The standard form of the Johnson–Cook model is given below:

(6) $$\sigma =\left[A+B\varepsilon^{n}\right]\left[1+C\ln{\dot{\varepsilon }}^{*}\right]\left[1-(\frac{T-Tref}{\;Tmelt\;-Tref}{)}^{m}\right]$$

Here, *A* denotes the initial yield strength [MPa], *B* is the strain-hardening coefficient [MPa], *n* is the strain-hardening exponent, *m* is the thermal softening exponent, *C* is the strain-rate strengthening coefficient, and ${\dot{\varepsilon }}^{*}$ is the dimensionless displacement rate denoted separately. Because all high-temperature tests in this study used the same displacement rate, the strain-rate effect can be treated as constant and the strain-rate term is omitted. The model then reduces to the following form.

(7) $$\sigma =(A+B\varepsilon^{n})(1-(\frac{T-Tref}{\;Tmelt\;-Tref}{)}^{m})$$

Here, $T_{ref}$ is 300.15 K and $T_{melt}$ is the melting temperature of PTFE.

**Figure 4:**
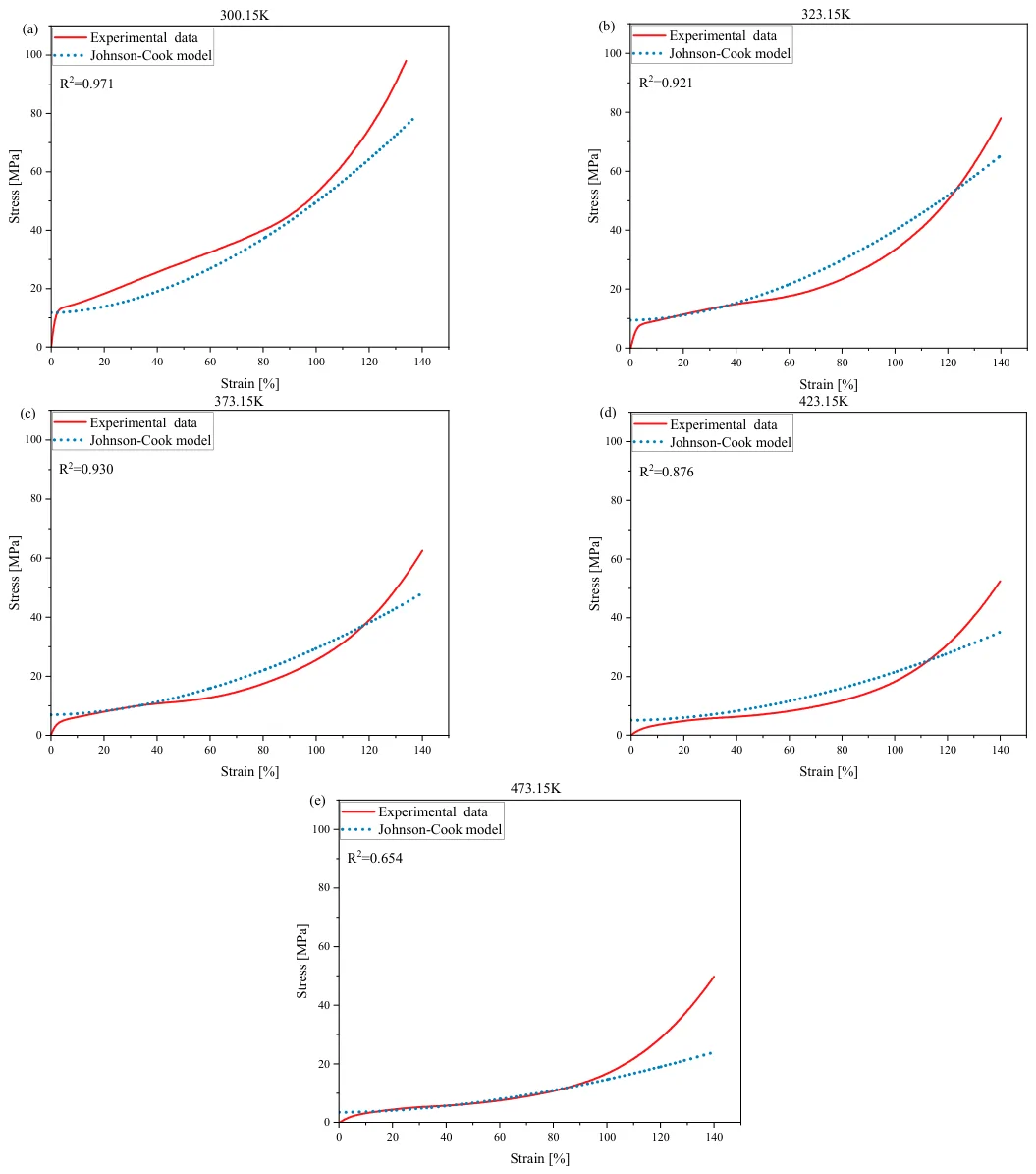
Comparison between Johnson–Cook model predictions and experimental true stress–strain curves: (**a**) 300.15 K, (**b**) 323.15 K, (**c**) 373.15 K, (**d**) 423.15 K, (**e**) 473.15 K.

Figure 4 compares the true stress–strain curves predicted by the Johnson–Cook model with the experimental results. Overall, the model captures the strain-hardening trend and the temperature-softening pattern reasonably well within the intermediate strain range of about 20 to 100 percent, but it deviates noticeably at both ends of the strain range. In the small-strain stage, from 0 to 20 percent, the Johnson–Cook model describes nonlinear plastic response through a power-law function and does not physically represent the initial linear elastic behavior. As a result, the predicted response in the elastic segment is nearly flat, which conflicts sharply with the rapid initial rise seen in the experimental curves. The underlying reason is that the power-law form of the Johnson–Cook model cannot accurately capture the initial response with a well-defined elastic modulus as strain approaches zero. In addition, the model fails to reflect the nonlinear accelerated hardening induced by the directional alignment of PTFE polymer chains at large deformation. These findings show that the Johnson–Cook model has inherent limitations for a material with multi-stage nonlinear behavior such as PTFE, and that a constitutive model with a more complete physical mechanism is needed for high-accuracy prediction across the full strain range. The specific parameter values for the fit are listed in Table 4.

The Ogden model is a representative framework within hyperelastic constitutive theory. By superposing multiple power functions of the principal stretch, it can effectively describe the nonlinear elastic response of rubber-like polymers at large deformation [26]. The strain energy function of the third-order Ogden model is given below.

(8) $$\sigma =\frac{\mu_{1}}{\alpha_{1}}\left(\lambda^{\alpha_{1}}-\lambda^{-\alpha_{1}/2}\right)+\frac{\mu_{2}}{\alpha_{2}}\left(\lambda^{\alpha_{2}}-\lambda^{-\alpha_{2}/2}\right)+\frac{\mu_{3}}{\alpha_{3}}\left(\lambda^{\alpha_{3}}-\lambda^{-\alpha_{3}/2}\right),\;\lambda =1+\varepsilon$$

Here, *μ*_1_, *μ*_2_, and *μ*_3_ denote the shear modulus contributions [MPa], *α*_1_, *α*_2_, and *α*_3_ the strain hardening or softening exponents, and *λ* the axial stretch ratio.

**Figure 5:**
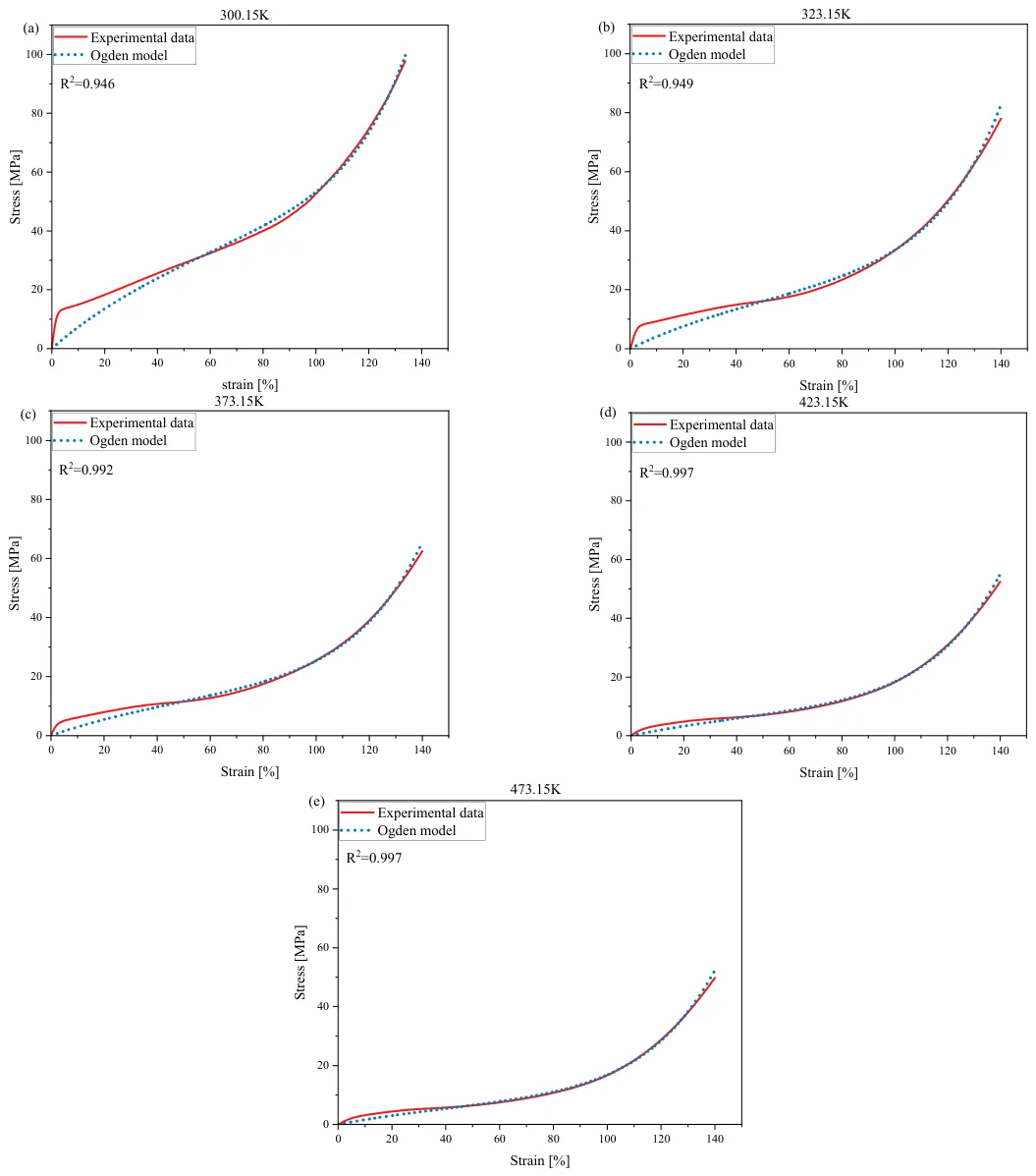
Comparison between the Ogden model predictions and experimental true stress–strain curves over the full strain range of 0 to 140 percent: (**a**) 300.15 K, (**b**) 323.15 K, (**c**) 373.15 K, (**d**) 423.15 K, (**e**) 473.15 K.

**Figure 6:**
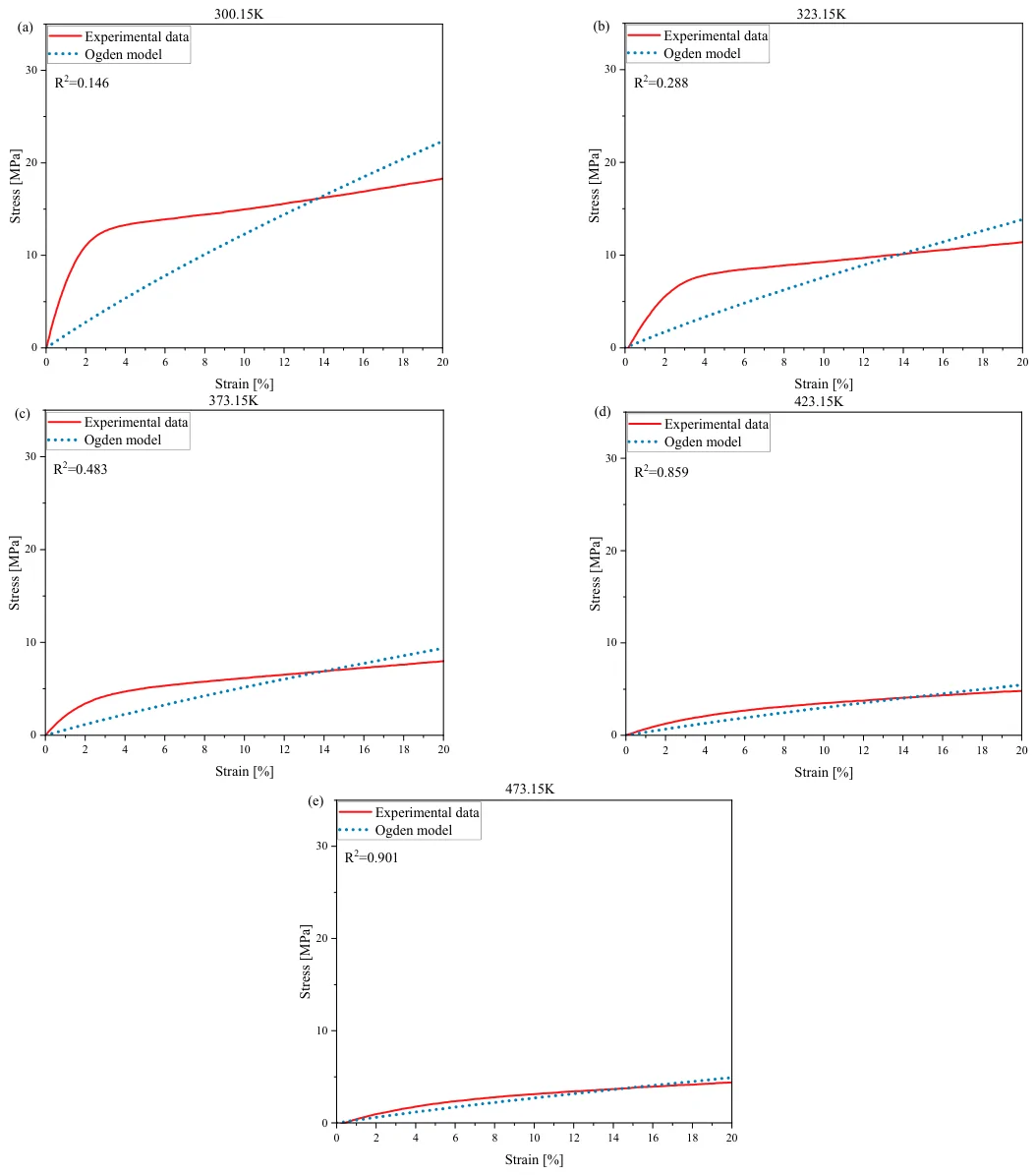
Comparison between Ogden model predictions and experimental true stress–strain curves over the small-strain range of 0 to 20 percent: (**a**) 300.15 K, (**b**) 323.15 K, (**c**) 373.15 K, (**d**) 423.15 K, (**e**) 473.15 K.

Figure 5 and Figure 6 compare the Ogden model predictions with the experimental results across the full strain range of 0 to 140 percent and the small-strain range of 0 to 20 percent, respectively. Over the full strain range, the Ogden model generally achieves high predictive accuracy and captures the overall stress–strain trend of PTFE well. As the temperature increases, the predicted curves become smoother and the accuracy improves further. This agrees with the experimental observation that the elastic segment of PTFE gradually disappears at high temperature, causing the deformation behavior to approach that of a purely elastic body, which suggests that the Ogden framework is well suited to the high-temperature regime. The specific parameter values for the fit are listed in Table 5.

In the small-strain range, however, the limitations of the Ogden model become evident. Below 373.15 K, and particularly at 300.15 K, the predicted initial elastic segment deviates substantially from the experimental values, with a coefficient of determination R^2^ of only 0.146, indicating almost no predictive capability. This arises because the Ogden model fundamentally describes reversible elastic deformation and cannot represent the yielding, plastic flow, and viscoplastic dissipation mechanisms of PTFE. Consequently, for a material such as PTFE that shows an elastic to plastic transition at room temperature, the small-strain prediction shows clear deviation. Overall, the Ogden model has value for fitting the full strain range, but its hyperelastic framework lacks a physically sound description of the elastic–plastic transition and irreversible plastic deformation in PTFE. Given the systematic deviation at low temperature and small strain, it is not suitable as the final constitutive model for PTFE across a wide temperature range. The specific parameter values for the fit are listed in Table 6.

The systematic evaluation of the Johnson–Cook and Ogden models shows that an ideal constitutive framework for PTFE must satisfy three requirements. It must accurately capture the initial linear elastic response, reasonably represent the transition through the yield plateau, and effectively describe the nonlinear strain hardening at large deformation. The ZWT nonlinear thermoviscoelastic constitutive model [27] satisfies the first two requirements to some extent and has been widely applied to describe the constitutive behavior of PTFE and its composites. Its expression is given below.

(9) $$\sigma =a_{1}\varepsilon +a_{2}\left(1-e^{-a_{3}\varepsilon }\right)$$

Here, *σ* is the true stress [MPa], *ε* is the true strain, dimensionless, and *a*_1_, *a*_2_, and *a*_3_ are fitting parameters.

**Figure 7:**
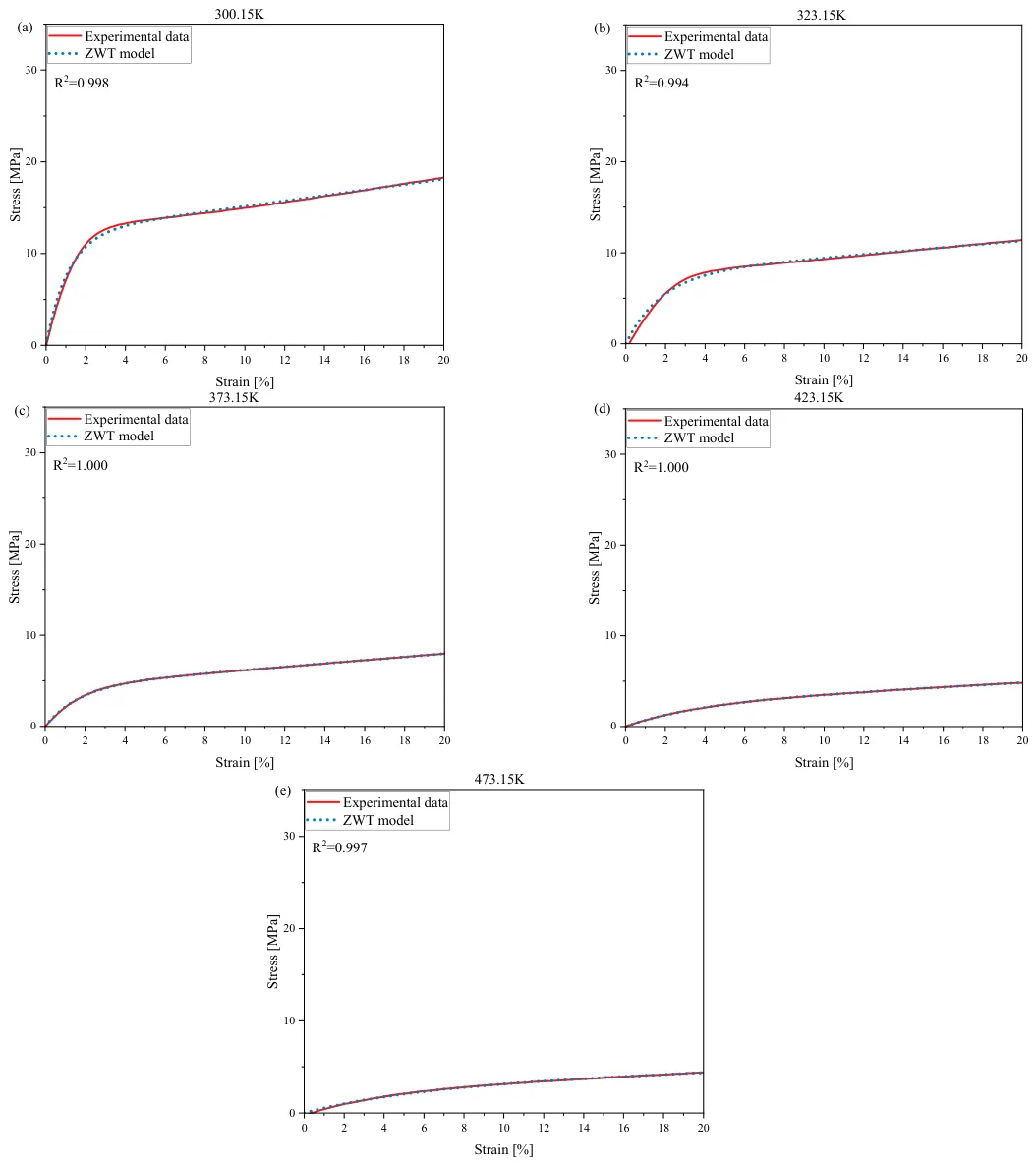
Comparison between ZWT model predictions and experimental true stress–strain curves over the small-strain range of 0 to 20 percent: (**a**) 300.15 K, (**b**) 323.15 K, (**c**) 373.15 K, (**d**) 423.15 K, (**e**) 473.15 K.

As shown in Figure 7, within the small-strain range of 0 to 20 percent, the ZWT model agrees closely with the experimental data across all temperatures from 300.15 to 473.15 K, with R^2^ values near 1. This indicates that the model statistically captures the initial elastic and viscoelastic transition behavior of PTFE well, and reasonably reflects the temperature-driven decrease in overall stress level. The model therefore shows strong physical validity and predictive reliability at small deformation. The specific parameter values for the fit are listed in Table 7.

**Figure 8:**
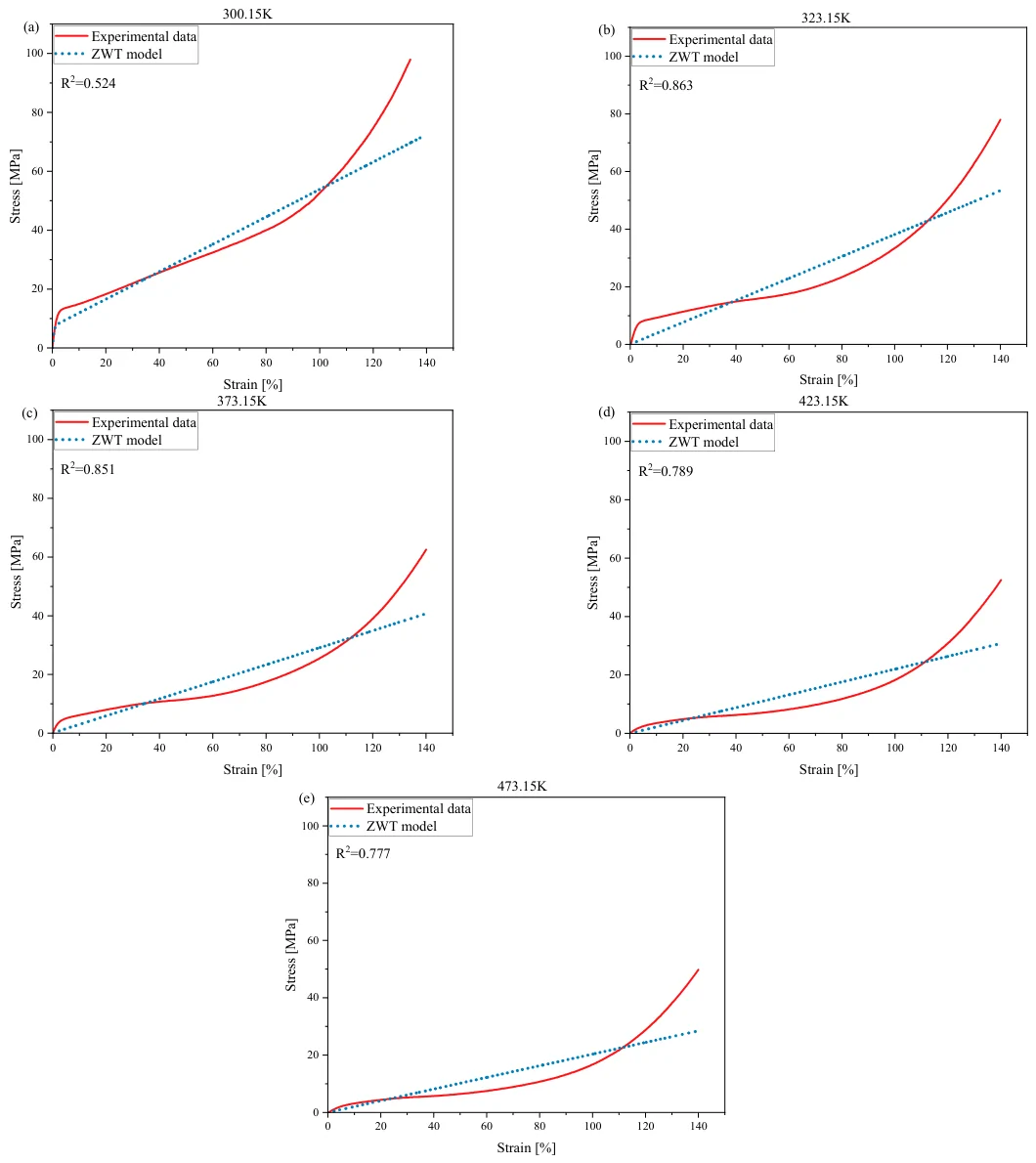
Comparison between ZWT model predictions and experimental true stress–strain curves over the full strain range of 0 to 140 percent: (**a**) 300.15 K, (**b**) 323.15 K, (**c**) 373.15 K, (**d**) 423.15 K, (**e**) 473.15 K.

When the strain enters the large-deformation range above 20 percent, however, the applicability of the ZWT model declines markedly. As shown in Figure 8, the experimental curves show a pronounced quadratic stress increase at large strain, while the ZWT model systematically underpredicts this behavior, with R^2^ values only ranging from 0.524 to 0.863, insufficient for an accurate description of large-deformation behavior. This limitation arises mainly because the second derivative of stress with respect to strain in the ZWT model is always negative [8], which prevents it from capturing the nonlinear accelerated hardening of the material at large strain. In addition, although higher temperature produces more pronounced stress softening, the experimental curves still show quadratic growth at large deformation, further highlighting the inadequacy of the ZWT model under complex thermomechanical coupling. These inherent limitations motivate the introduction of a higher-order correction term that preserves the strengths of the ZWT model at small deformation while achieving a unified description across the full strain range and a wide temperature range. The specific parameter values for the fit are listed in Table 8.

### 3.3. TPS Thermomechanically Coupled Constitutive Model

To overcome the inherent limitations of the ZWT model at large deformation, a prior study introduced a higher-order equilibrium stress correction and proposed the TPS model [9], expressed as follows.

(10) $$\sigma =\sigma_{m}\left\{1-\exp\left[-\left(m\varepsilon +0.5m^{2}\varepsilon^{2}\right)\right]\right\}+E_{t}\varepsilon +\exp\left[\alpha \varepsilon +\beta \right]$$

Here, $\sigma_{m}$ is the yield stress [MPa], m is the ratio of the initial elastic modulus to the yield stress, $E_{t}$ is the tangent modulus after yielding, α is the growth coefficient, and *β* is the offset coefficient. This model decomposes the macroscopic stress response of PTFE into a progressive growth term, a linear growth term, and an exponential growth term, corresponding respectively to microscopic lattice elastic deformation, slip-induced plastic deformation in crystalline regions, and chain reorientation hardening at large deformation. The TPS model achieves high predictive accuracy across the entire strain range, from initial loading to fracture, and across a wide low-temperature range extending from liquid nitrogen temperature to room temperature. It also establishes a unified physical connection between the model structure and the macroscopic mechanical behavior and microscopic crystalline features of the material.

The parameters of the original TPS model are constants, however, and the model applies only to a fixed temperature. Its validity under high-temperature conditions has not been verified. To extend the model across a wide temperature range, parameter-temperature evolution equations were developed based on Arrhenius thermal activation theory [28,29]. The central idea of the Arrhenius equation is that the activation energy required for polymer chain segments to overcome local energy barriers and undergo slip or deformation determines a rate that increases exponentially with temperature.

(11) $$\dot{\varepsilon }\propto \exp\left(-\frac{Q}{RT}\right)$$

Here, *Q* is the apparent activation energy of material deformation [J/mol], *R* is the molar gas constant (8.314 J/[mol·K]), and *T* is the absolute thermodynamic temperature [K]. At a fixed displacement rate, the characteristic stress that the external field must supply for the material to yield or flow macroscopically is inversely related to this thermally activated rate. As a result, the stiffness and yield parameters of the material show a positive exponential relationship with temperature *T*.

(12) $$P(T)=A\cdot \exp\left(\frac{Q}{RT}\right)$$

Here, *P(T)* is the mechanical characteristic parameter at temperature *T*, and *A* is the pre-exponential factor. Based on this theoretical framework, temperature evolution equations were established for each parameter, as follows.

(I) Yield stress parameter ${(\sigma }_{m}(T))$ and tangent modulus parameter $\left(E_{t}(T)\right)$

These two parameters directly reflect the resistance to disentanglement arising from van der Waals forces and lattice constraints between PTFE molecular chains. As temperature *T* rises, thermal motion intensifies and the external stress required to overcome these barriers decreases. Following classical polymer rheology, baseline values are defined under a reference state. To remove physical dimensions and improve the numerical stability of the fitting, the reference temperature, *T_ref_* = 300.15 K, is introduced.

(13) $$\sigma_{m}(T)=\sigma_{m,0}\cdot \exp\left[\frac{Q_{\sigma }}{R}\left(\frac{1}{T}-\frac{1}{T_{ref}}\right)\right]+\sigma_{m,\mathrm{residual}}$$

Considering that PTFE retains network resistance even at very high temperature and does not become a fully liquid state, a residual stress correction term $\sigma_{m,residual}$ is introduced.

(14) $$\sigma_{m}(T)=A_{1}\cdot \exp\left(\frac{B_{1}}{T}\right)+C_{1}$$

Similarly, the tangent modulus parameter $\left(E_{t}(T)\right)$ shows purely exponential decay toward zero at high temperature, as seen in Figure 3, so it follows the standard Arrhenius form.

(15) $$E_{t}(T)=A_{2}\cdot \exp\left(\frac{B_{2}}{T}\right)$$

where $B_{i}=\frac{Q_{i}}{R}.$

(II) Ratio of the initial elastic modulus to yield stress (m(T))

The parameter (m(T)) strongly influences the curvature that characterizes the transition from the elastic stage to plastic flow. The experimental results show that it decreases sharply with rising temperature and stabilizes at high temperature. This indicates that the transition mechanism is also governed by a thermally activated rate, described using an Arrhenius structure.

(16) $$m(T)=A_{3}\cdot \exp\left(\frac{B_{3}}{T}\right)+C_{3}$$

(III) Correction of the stress growth parameters (α(T)) and (β(T))

Because the orientation stretching and slip disentanglement of PTFE molecular chains at large strain are influenced by temperature-driven phase transitions, α(T) and β(T) show non-monotonic, U-shaped or inverted U-shaped variation. A single Arrhenius pathway cannot directly describe this non-monotonic trajectory. To remain consistent with the activation energy framework while accommodating this behavior, a dual activation energy competition model or a polynomial correction is typically used. Here, both parameters are expressed as quadratic polynomials of temperature.

(17) $$\alpha (T)=A_{4}T^{2}+B_{4}T+C_{4}$$

(18) $$\beta (T)=A_{5}T^{2}+B_{5}T+C_{5}$$

Substituting these results back into the original TPS constitutive equation yields the final full-temperature thermomechanically coupled constitutive model for PTFE.

(19) $$\left\{\begin{array}{l} \begin{array}{l} \sigma (\varepsilon ,T)=\sigma_{m}(T)\left\{1-\exp\left[-\left(m(T)\varepsilon +0.5m{\left(T\right)}^{2}\varepsilon^{2}\right)\right]\right\}\\ +E_{t}(T)\varepsilon +\exp\left[\alpha (T)\varepsilon +\beta (T)\right] \end{array}\\ \sigma_{m}(T)=A_{1}\exp\left(\frac{B_{1}}{T}\right)+C_{1}\\ E_{t}(T)=A_{2}\exp\left(\frac{B_{2}}{T}\right)\\ m(T)=A_{3}\exp\left(\frac{B_{3}}{T}\right)+C_{2}\\ \alpha (T)=A_{4}T^{2}+B_{4}T+C_{3}\\ \beta (T)=A_{5}T^{2}+B_{5}T+C_{4} \end{array}\right.$$

The yield stress parameter ${(\sigma }_{m}(T))$, the tangent modulus parameter $\left(E_{t}(T)\right)$, and the ratio parameter (m(T)) all show exponential decay as temperature rises, reflecting the intensified thermal motion of polymer chain segments and the thermal softening of the matrix. The parameters (α(T))  and (β(T)), which characterize the secondary stress growth at large deformation, are represented by quadratic polynomials in temperature, describing the mechanical behavior of the material during the large-deformation orientation stage at different temperatures.

**Figure 9:**
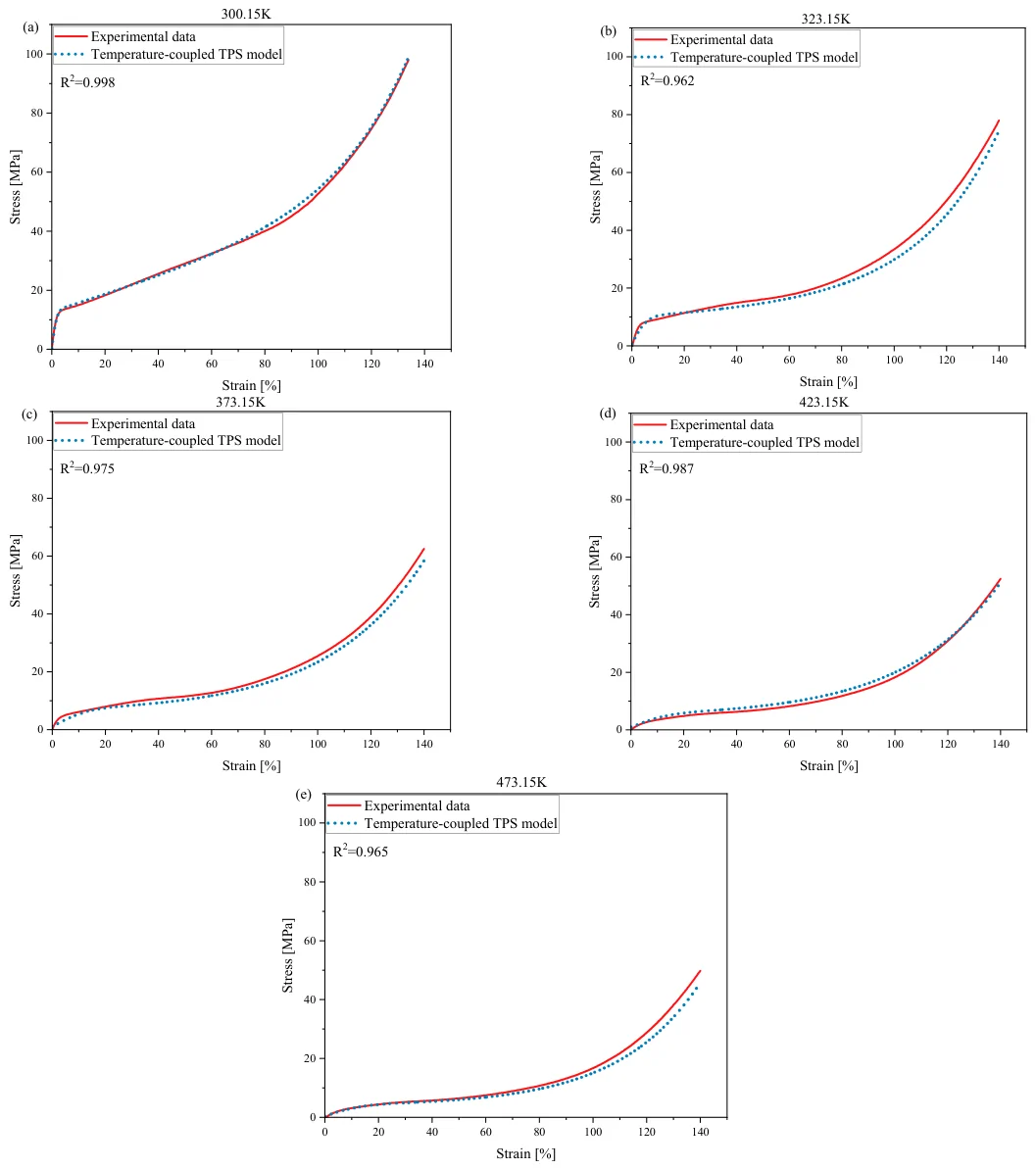
Comparison between predictions of the temperature-coupled TPS model and experimental true stress–strain curves: (**a**) 300.15 K, (**b**) 323.15 K, (**c**) 373.15 K, (**d**) 423.15 K, (**e**) 473.15 K.

Figure 9 presents a comprehensive comparison between the predictions of the temperature-coupled TPS model and the experimental data. At 300.15, 323.15, 373.15, 423.15, and 473.15 K, the predicted values agree closely with the experimental data across the entire deformation range, with R^2^ values exceeding 0.96 at every temperature. Compared with the Johnson–Cook and Ogden models, the TPS model accurately captures the small-strain elastic stage, the intermediate yield plateau, and the large-deformation strain-hardening stage. Compared with the ZWT model, it successfully represents the secondary stress growth at large strain, overcoming a fundamental limitation of the ZWT model. Taken together, these comparisons show that the temperature-coupled TPS model offers the best predictive capability for the mechanical behavior of PTFE across a wide temperature range and the full strain range, providing a reliable theoretical tool for the engineering design and service life assessment of this material under extreme operating conditions. The specific parameter values for the fit are listed in Table 9.

### 3.4. Molecular Dynamics Simulation

The temperature-dependent evolution of *σ_m_*(*T*) and *E_t_*(*T*) in Section 3.3 was interpreted through the lens of Arrhenius thermal activation theory, which assumes that increasing temperature accelerates the rate at which polymer chain segments overcome local energy barriers. This assumption implies two testable molecular-scale consequences: an increase in the space available for segmental motion (free volume), and an increase in the actual mobility of chain segments (diffusion). Molecular dynamics simulations were therefore performed to test both of these implications directly.

**Figure 10:**
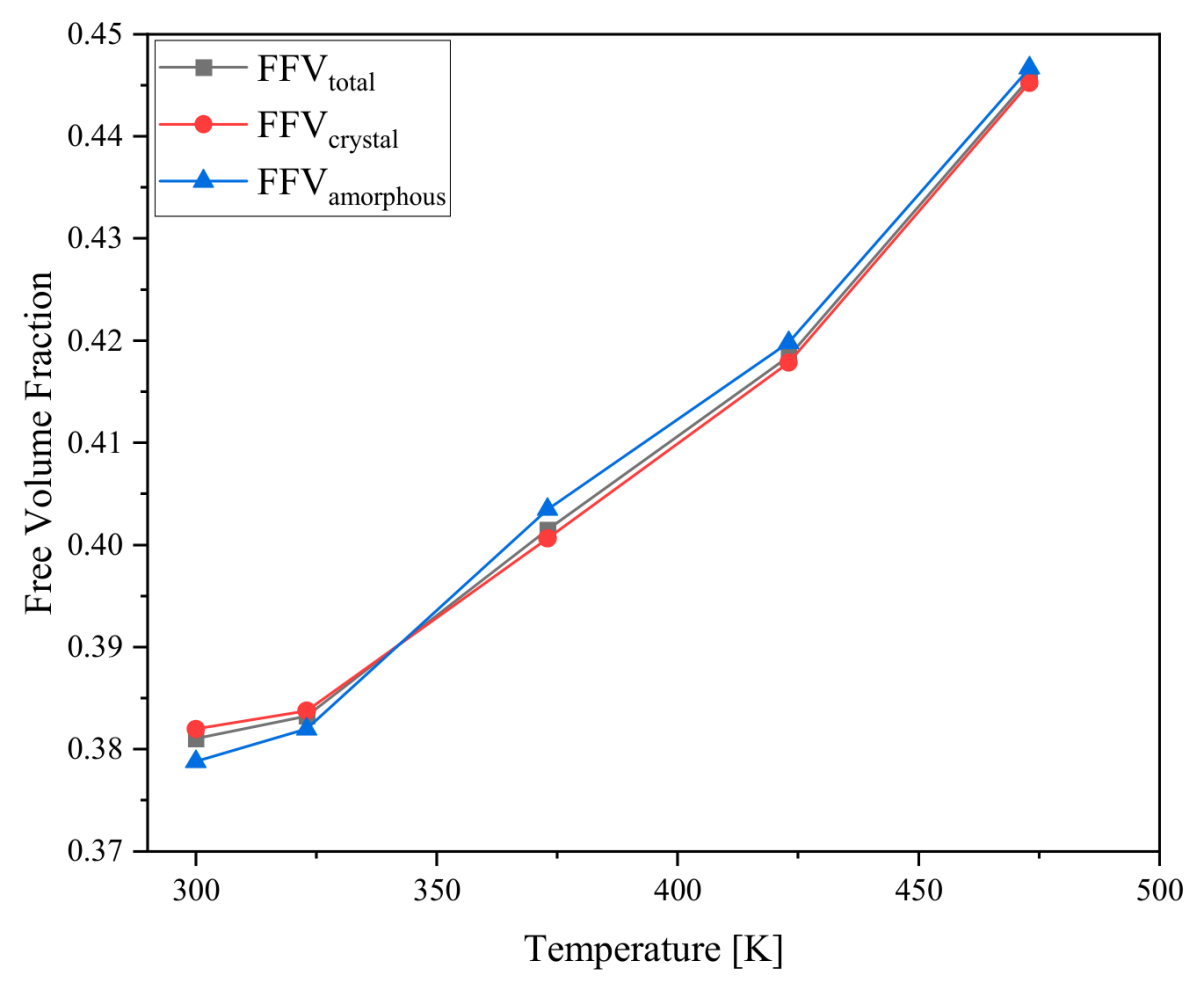
Variation in free volume fraction with temperature.

Figure 10 shows the variation in the total free volume fraction (FFV_total_) and the free volume fractions of crystalline and amorphous regions of PTFE with temperature from 300.15 K to 473.15 K. The total free volume fraction (FFV_total_) increases from 0.381 at 300.15 K to 0.446 at 473.15 K, an increase of approximately 17%. Within the studied temperature range, the free volume fractions of the crystalline and amorphous regions remain close to each other, with the amorphous region showing a slightly faster increase at 17.8%, compared to 16.7% for the crystalline region. This relatively small absolute difference is consistent with the specific crystal structure employed by PTFE: the crystalline regions in the model are constructed as a pseudo-hexagonal I-phase structure, a disordered helical lattice in which the chains maintain long-range translational order along the chain axis but undergo continuous rotation around the chain axis. Therefore, its chain packing is inherently looser than that of completely ordered, densely packed polymer crystals such as polyethylene. The large van der Waals radii of the fluorine substituents on the PTFE backbone further endow the PTFE crystalline regions with a relatively high intrinsic free volume, thus narrowing the gap between the free volume fractions of crystalline and amorphous regions compared to densely packed semi-crystalline polymers. This also indicates that the relatively loose chain packing of the amorphous regions allows for greater configurational adjustment under thermal expansion, while the crystalline regions, constrained by ordered packing, exhibit slightly lower thermal sensitivity of their free volume. The increase in free volume with increasing temperature reflects the weakening of intermolecular constraints between chain segments, providing more space for chain segment movement. This trend is consistent with the mechanism of the decrease in flow stress at high temperatures observed experimentally in Section 3.1.

**Figure 11:**
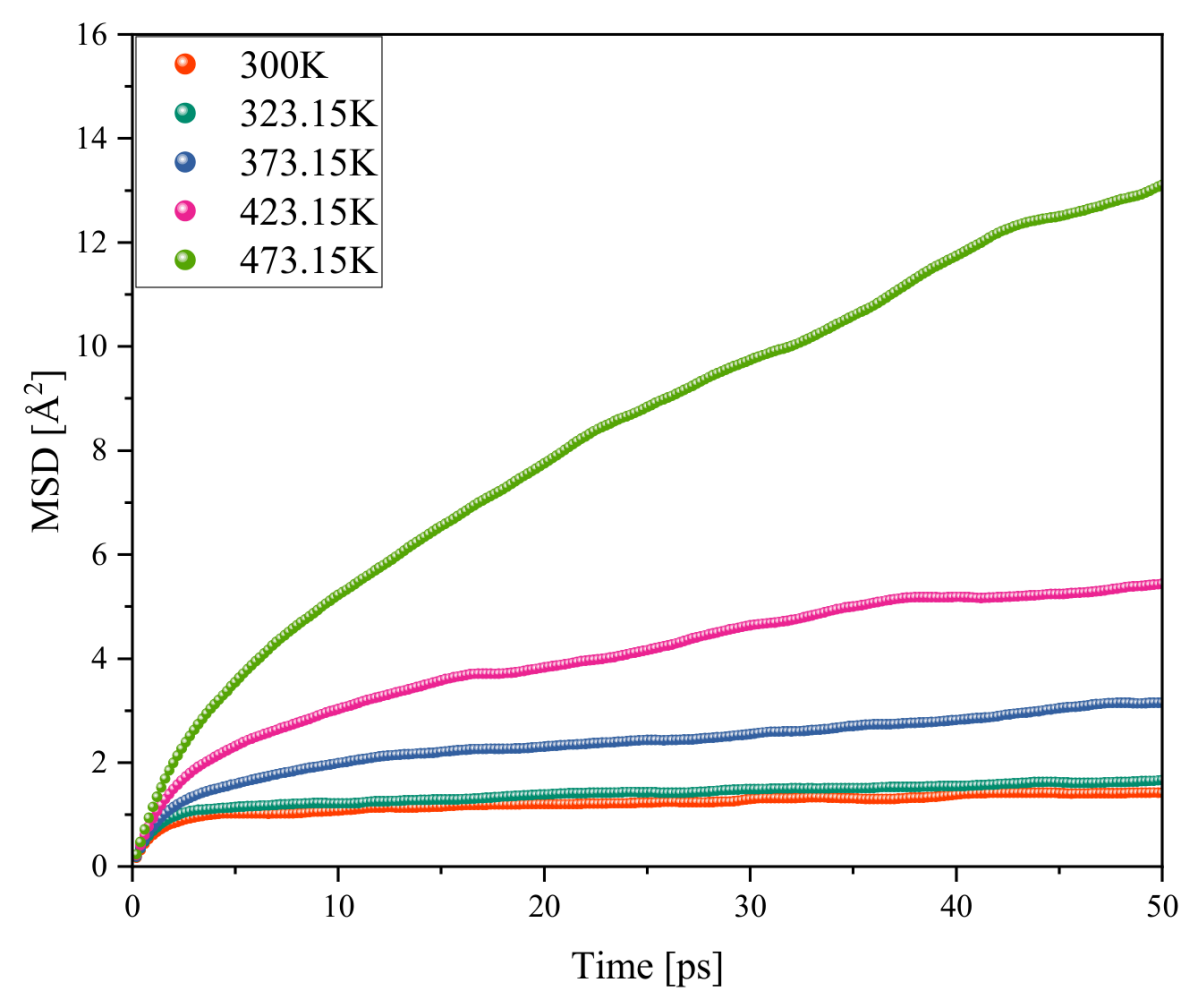
Variation in PTFE segmental mean-square displacement with temperature.

Figure 11 shows the mean-square displacement of the PTFE chain centroid over time at different temperatures. The MSD curves show ballistic behavior at short times, followed by a diffusive regime, and the slope of the diffusive regime increases with temperature. The MSD at 200 ps increases from 1.4 Å^2^ at 300.15 K to 13.1 Å^2^ at 473.15 K, showing that elevated temperature substantially enhances the translational mobility of chain segments. This result corroborates the reduced steric hindrance associated with increased free volume. Using the Einstein relation applied to the long-time linear region of the MSD curves, the diffusion coefficient increases from 1.40 × 10^−7^ cm^2^/s at 300.15 K to 3.00 × 10^−6^ cm^2^/s at 473.15 K.

**Figure 12:**
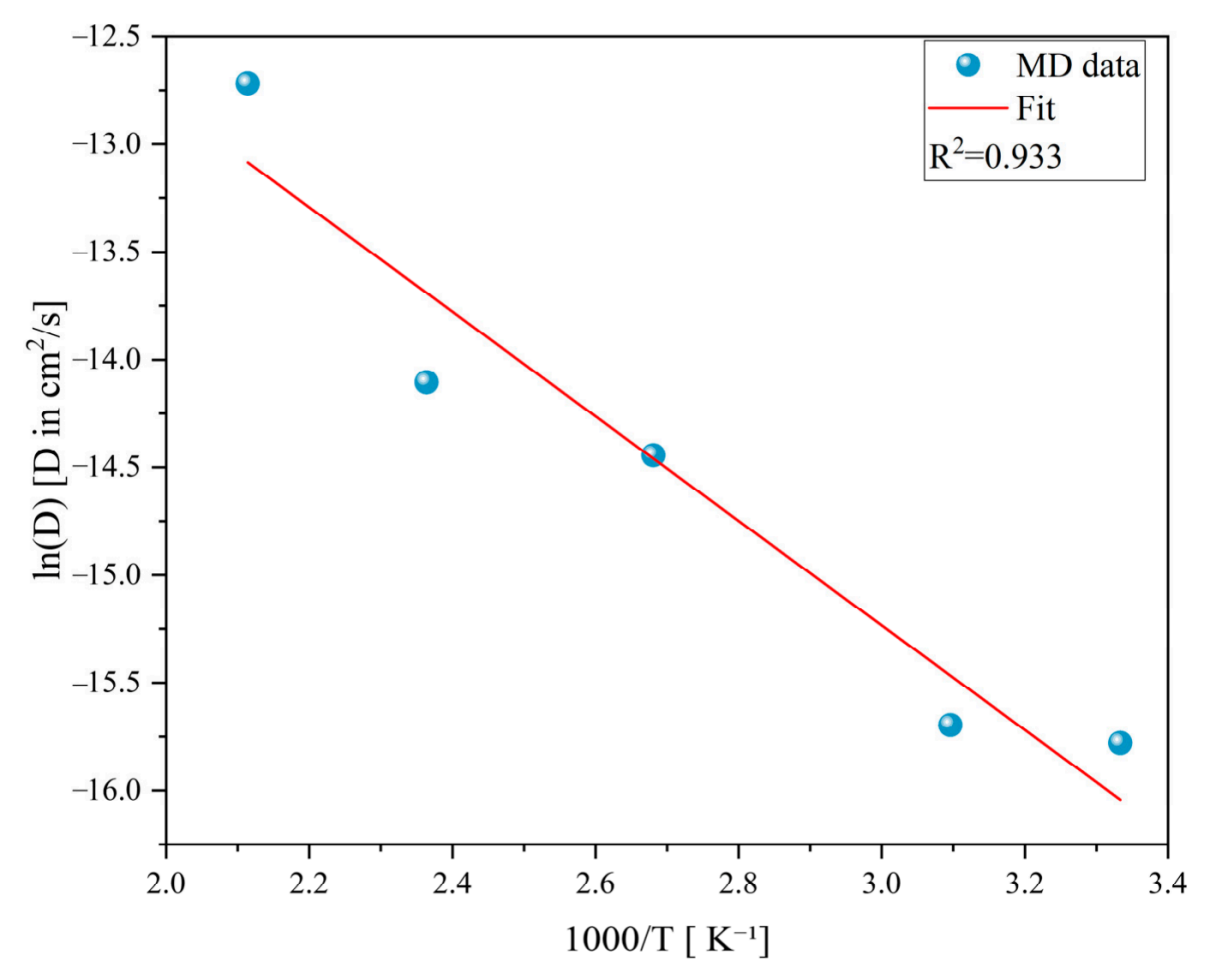
Arrhenius fit of the diffusion coefficient.

Figure 12 shows the Arrhenius fit of the diffusion coefficient *D*. The natural logarithm of *D* shows a good linear relationship with 1000/T, with a goodness of fit R^2^ of 0.933. The clear Arrhenius dependence of the diffusion coefficient mirrors the functional form adopted for *σ_m_*(T) and *E_t_*(T) in Section 3.3, lending qualitative support to the use of the same mathematical framework at both the molecular and macroscopic scales. The apparent activation energy Q_MD_ is 20.17 kJ/mol, and the pre-exponential factor *D_0_* is 3.51 × 10^−4^ cm^2^/s. This activation energy is close to the range reported for the *β* relaxation process in fluoropolymers [30,31], suggesting that the segmental motion captured in the simulation corresponds mainly to local conformational transitions within the amorphous region rather than long-range cooperative diffusion. This result also provides molecular-level support for the Arrhenius description of temperature-dependent constitutive parameters presented in Section 3.3.

The analysis of free volume and segmental mobility offers a molecular-level explanation for the thermal softening of PTFE observed in experiments. On one hand, rising temperature increases the free volume fraction, which lowers the density of van der Waals contacts between chains and reduces the energy required for relative segmental displacement. On the other hand, segmental mobility increases exponentially with temperature, allowing segments to relax stress more readily through conformational transitions. Together, these two effects produce the macroscopically observed decrease in flow stress with rising temperature, consistent with the molecular-level changes in free volume and segmental dynamics [32].

**Figure 13:**
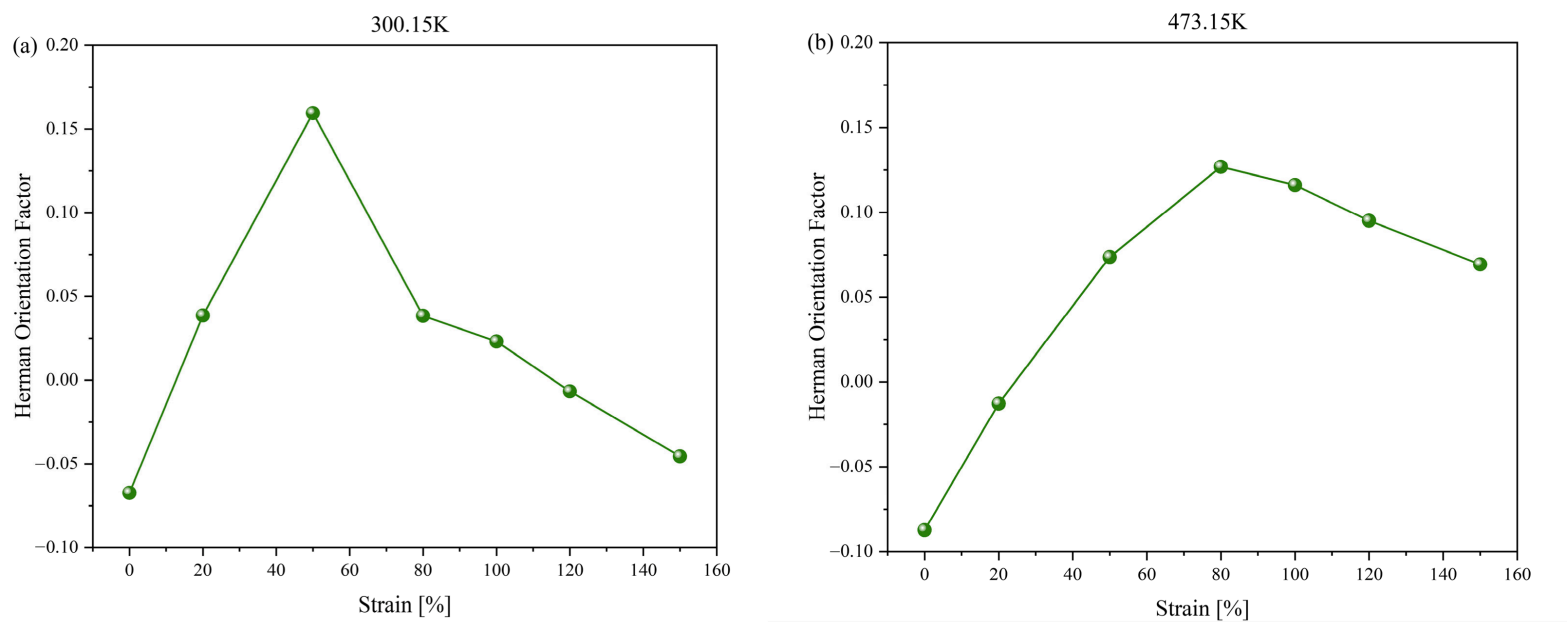
Variation in the Herman orientation factor with strain during tension: (**a**) 300.15 K and (**b**) 473.15 K.

Figure 13 shows the variation in the Herman orientation factor with strain at 300.15 and 473.15 K. At both temperatures, the orientation factor first increases and then decreases with increasing strain. At 300.15 K, it reaches a peak value of 0.160 near 50 percent strain, and at 473.15 K, it reaches a peak value of 0.127 near 80 percent strain, followed by a decline of varying degree at both temperatures. The orientation factor shifts from an initially negative value to a positive value, indicating that the chain segments transition from a slightly perpendicular orientation relative to the tensile direction in the initial semicrystalline structure toward alignment along the tensile direction. This is consistent with the classical understanding that amorphous segments and crystalline lamellae progressively reorient along the loading direction during the deformation of polymer materials [33,34].

Orientation saturation occurs at a smaller strain at 300.15 K than at 473.15 K, possibly because the lower segmental mobility at low temperature limits further chain orientation beyond moderate strain [35]. The decline in the orientation factor after the peak may reflect partial disorientation and local rearrangement of chain segments during continued large deformation, a phenomenon also reported in the large-deformation behavior of other semicrystalline polymers [36,37].

**Figure 14:**
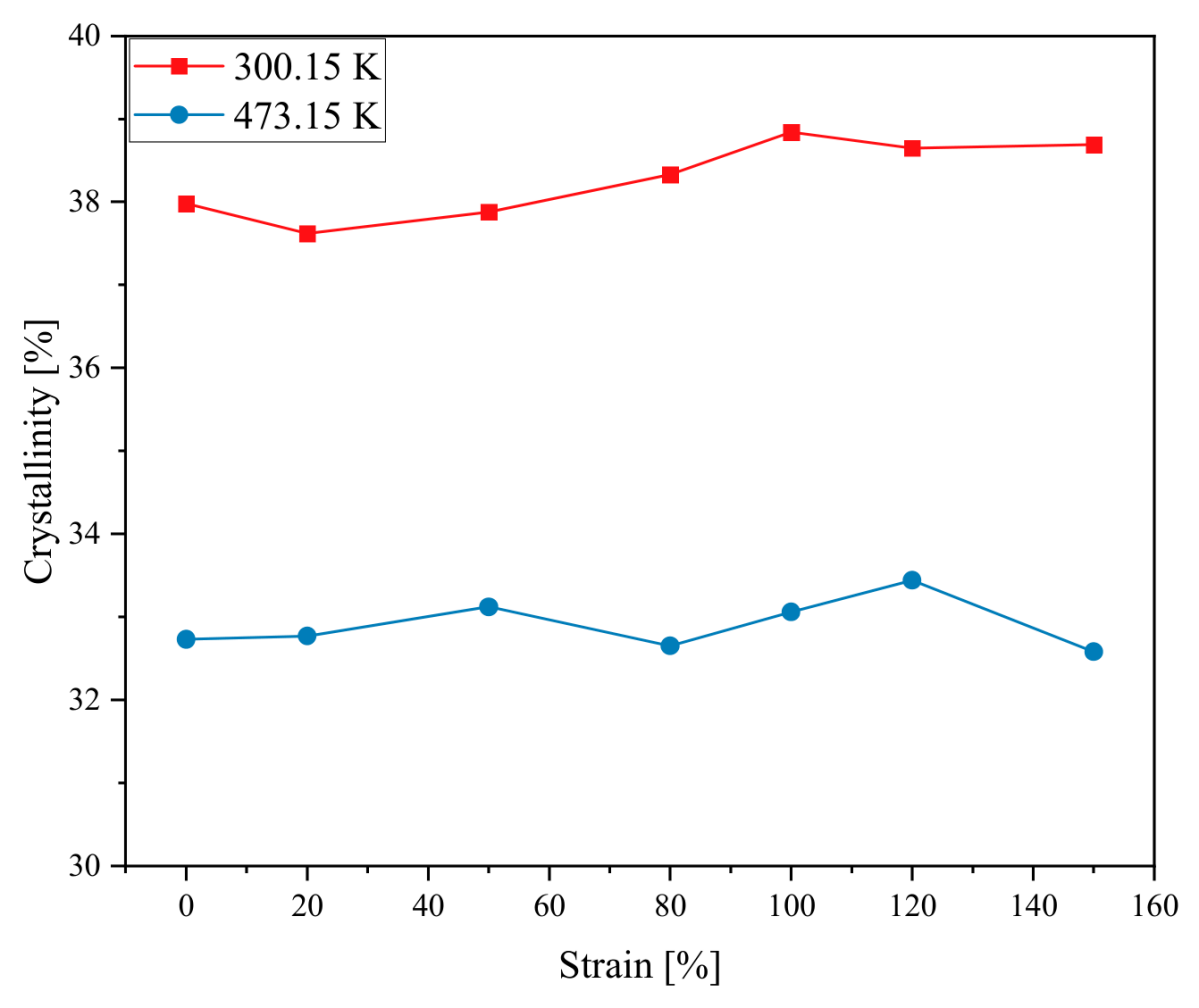
Variation in crystallinity with strain during tension.

Figure 14 shows the variation in crystallinity with strain at 300.15 and 473.15 K. Across the 0 to 150 percent strain range, crystallinity remains relatively stable at both temperatures, fluctuating between 37.6 and 38.8 percent at 300.15 K and between 32.6 and 33.4 percent at 473.15 K, with no evidence of strain-induced amorphization. This indicates that the crystalline structure remains essentially intact within the deformation range simulated here, and that strain is accommodated mainly through affine deformation of the crystalline lamellae, interlamellar shear slip, and the stretching and orientation of amorphous tie chains. The lower absolute crystallinity at 473.15 K compared with 300.15 K mainly reflects differences in the initial thermal equilibrium state at the two temperatures rather than a deformation-induced change in crystallinity.

Taken together, the analyses of free volume, segmental mobility, chain orientation, and crystallinity provide a coherent picture of the molecular mechanisms underlying the tensile deformation of PTFE. At small strain, both the crystalline and amorphous regions contribute to elastic deformation. As strain increases, the amorphous tie chains progressively stretch and align along the loading direction, and the crystalline lamellae rotate through intralamellar slip, while crystallinity remains essentially unchanged throughout this process. This indicates that deformation proceeds mainly through the rearrangement of existing structural units rather than through the disruption of order in the amorphous region. The continued increase in chain orientation with strain provides the structural basis for macroscopic strain hardening, since more highly oriented chains can bear greater load along the tensile direction.

Taken together, the wide-temperature tensile tests reveal the macroscopic manifestation of thermal softening and enhanced ductility; the temperature-coupled TPS model provides a quantitative phenomenological description of this behavior through physically motivated, thermally activated parameters; and the molecular dynamics simulations independently confirm that the same thermally activated character emerges naturally from the temperature dependence of free volume and segmental mobility at the molecular scale. This convergence across three levels of description—macroscopic, phenomenological, and molecular—supports the physical validity of the proposed constitutive framework.

## 4. Conclusions

This study combines wide-temperature-range uniaxial tensile tests, a temperature-dependent TPS constitutive model, and molecular dynamics simulation to investigate the mechanical response and thermal softening mechanism of PTFE tube material under large deformation. The main conclusions are as follows.

(1) The tensile mechanical properties of PTFE along the axial and circumferential directions differ only slightly, indicating that the material does not show pronounced mechanical anisotropy. Within the quasi-static displacement rate range of 20 to 100 mm/min, displacement rate has a limited effect on the strength and ductility of the material, indicating relatively low-rate sensitivity.
(2) Temperature is the dominant factor governing the tensile behavior of PTFE. As the test temperature rises from 300.15 to 473.15 K, the flow stress decreases markedly, while the ductility increases substantially. Elevated temperature reduces the resistance of the material to plastic flow while enhancing its capacity for large deformation, producing pronounced thermal softening and toughening.
(3) The Johnson–Cook model cannot accurately describe the secondary stress growth of PTFE at large deformation. The Ogden model has limited applicability to the yield plateau and the temperature-dependent response. The ZWT model captures some viscoelastic features but still shows deviation across the full tensile response over a wide temperature range. By contrast, the temperature-dependent TPS model, built on Arrhenius theory, describes the initial elastic stage, the yield plateau, and the subsequent nonlinear strain hardening well, achieving coefficients of determination above 0.96 across the tested temperature range.
(4) Molecular dynamics simulations show that as temperature increases, both the free volume fraction and the segmental mobility of PTFE increase markedly, indicating that elevated temperature facilitates chain conformational adjustment and plastic flow. Meanwhile, as tensile strain increases, the degree of chain orientation continues to rise while the crystallinity of the crystalline region remains essentially stable. These results indicate that strain hardening at large deformation is associated mainly with the stretching and orientation of amorphous chain segments and the rearrangement of crystalline lamellae, rather than with substantial disruption of the crystalline structure. This molecular-scale evolution is consistent with the thermal softening and strain hardening observed experimentally, providing a microscopic mechanistic explanation for the macroscopic mechanical response.

The temperature-dependent TPS model established in this study can serve as a verification tool for the finite element structural design and life assessment of components such as PTFE pipes over a wide service temperature range. It is particularly suitable for predicting the large deformation response of materials near the thermal softening initiation temperature. Future study can expand this framework in the following two directions: (1) extending molecular dynamics analysis to cyclic loading and multiaxial loading conditions to better fit the actual service conditions of expansion joints; and (2) incorporating long-term creep and stress relaxation behavior into the temperature-coupled framework to support life prediction under continuous thermomechanical coupling loads.

## Figures and Tables

**Table 1:** Room-temperature uniaxial tensile test conditions for PTFE.

Sampling Direction	Displacement Rate [mm/min]	Test Temperature [K]
Circumferential	20	300.15
Circumferential	50	300.15
Circumferential	100	300.15
Longitudinal	20	300.15
Longitudinal	50	300.15
Longitudinal	100	300.15

**Table 2:** Mechanical property parameters of PTFE specimens under different tensile conditions.

Sampling Direction	Displacement Rate [mm/min]	Elastic Modulus [MPa]	Yield Strength [MPa]	Tensile Strength [MPa]	Fracture Elongation [%]
Circumferential	20	$861_{-11}^{+14}$	$12.73_{-0.23}^{+0.18}$	$25.76_{-0.17}^{+0.22}$	$274_{-5}^{+6}$
Circumferential	50	$845_{-18}^{+18}$	$13.09_{-0.13}^{+0.13}$	$25.89_{-0.21}^{+0.12}$	$277_{-7}^{+5}$
Circumferential	100	$839_{-19}^{+23}$	$13.75_{-0.40}^{+0.30}$	$26.42_{-0.67}^{+0.46}$	$281_{-4}^{+4}$
Longitudinal	20	$839_{-8}^{+11}$	$13.36_{-0.30}^{+0.22}$	$26.16_{-0.33}^{+0.34}$	$263_{-2}^{+3}$
Longitudinal	50	$861_{-11}^{+9}$	$13.79_{-0.13}^{+0.19}$	$26.77_{-0.31}^{+0.36}$	$279_{-10}^{+10}$
Longitudinal	100	$851_{-9}^{+10}$	$13.48_{-1.12}^{+0.84}$	$26.80_{-0.36}^{+0.32}$	$277_{-9}^{+12}$

**Table 3:** High-temperature uniaxial tensile test conditions for PTFE.

Sampling Direction	Displacement Rate [mm/min]	Test Temperature [K]
Circumferential	50	323.15
Circumferential	50	373.15
Circumferential	50	423.15
Circumferential	50	473.15
Longitudinal	50	323.15
Longitudinal	50	373.15
Longitudinal	50	423.15
Longitudinal	50	473.15

**Table 4:** Fitted parameters of the Johnson–Cook model.

*A* [MPa]	*B* [MPa]	*n*	*m*
11.70	43.55	2.00	0.52

**Table 5:** Fitted parameters of the Ogden model over the full strain range (0–140%).

*μ*_1_ [MPa]	*μ*_2_ [MPa]	*μ*_3_ [MPa]	*α*_1_	*α*_2_	*α*_3_
0.001	21.676	0.056	1.570	−2.338	10.172

**Table 6:** Fitted parameters of the Ogden model over the small-strain range (0–20%).

*μ*_1_ [MPa]	*μ*_2_ [MPa]	*μ*_3_ [MPa]	*α*_1_	*α*_2_	*α*_3_
0.001	23.441	0.001	0.104	−5.751	2.326

**Table 7:** Fitted parameters of the ZWT model over the small-strain range (0–20%).

*a*_1_	*a*_2_	*a*_3_
17.93	4.39	59.07

**Table 8:** Fitted parameters of the ZWT model over the full strain range (0–140%).

*a*_1_	*a*_2_	*a*_3_
38.06	0.1	755.72

**Table 9:** Fitted parameters of the temperature-coupled TPS model.

*A*_1_	*A*_2_	*A*_3_	*A*_4_	*A*_5_	*B*_1_	*B*_2_	*B*_3_	*B*_4_	*B*_5_	*C*_1_	*C*_2_	*C*_3_	*C*_4_
5.42 × 10^−2^	2.74 × 10^−10^	1.16 × 10^−7^	1.04 × 10^−4^	−1.45 × 10^−4^	1572.35	7601.36	5939.04	−0.08	0.11	2.28	7.17	19.12	−21.88

## Data Availability

The original contributions presented in this study are included in the article. Further inquiries can be directed to the corresponding authors.

## Abbreviations

The following abbreviations are used in this manuscript.

PTFE	Polytetrafluoroethylene
TPS	Three-part superposition model
ZWT	Zhu–Wang–Tang models
MD	Molecular dynamics
LAMMPS	Large-scale Atomic/Molecular Massively Parallel Simulator
NVT	Constant number of particles, volume, and temperature (ensemble)
NPT	Constant number of particles, pressure, and temperature (ensemble)
MSD	Mean-square displacement
FFV	Fractional free volume
R^2^	Coefficient of determination
FFV_total_	Total free volume fraction
FFV_crystal_	Free volume fraction of the crystalline regio
FFV_amorphous_	Free volume fraction of the amorphous region
